# Estimating food availability and self-reliance in island territories: Puerto Rico as a case study

**DOI:** 10.3389/fnut.2025.1622876

**Published:** 2025-08-19

**Authors:** Nayla Bezares, Chad Fisher, Carol E. Ramos-Gerena, William Suarez-Gomez, Sean B. Cash, Thomas Nemecek, Nicole Tichenor Blackstone

**Affiliations:** ^1^Gerald J. and Dorothy R. Friedman School of Nutrition Science and Policy, Tufts University, Boston, MA, United States; ^2^School of Architecture and Planning, University at Buffalo-State University of New York, Buffalo, NY, United States; ^3^Health Equity, Administration & Technology Department, City University of New York- Lehman College, Bronx, NY, United States; ^4^Life Cycle Assessment Group, Sustainability Assessment and Agricultural Management, Agroscope, Zürich, Switzerland

**Keywords:** self-reliance, food availability, dietary recommendations, food imports, Puerto Rico, Small Island Developing States (SIDS), territories

## Abstract

**Introduction:**

In Small Island Developing States (SIDS), limited statistical capacity and reliance on imports hinder comprehensive assessments of food systems. For island territories, this issue is more pronounced as food production data are disaggregated, inconsistent, and scarce. Most non-independent territories within the SIDS designation are not included in international food availability datasets, and local datasets are not readily available. Increased food self-reliance has been proposed to enhance food nutrition security and sovereignty across SIDS. Puerto Rico, an island territory of the United States, is highly reliant on food imports.

**Methods:**

Using local import, export, and production records from fiscal years 2017–2019, combined with representative food loss and waste estimates, we developed datasets quantifying food availability and self-reliance metrics for Puerto Rico as a case study. A novel data crosswalk, adaptable to other island territories, supports the replication of this approach.

**Results:**

Oils, grains, and protein foods had the highest per-capita availability. Agricultural self-reliance was highest for dairy (95%), fruits (47%), and vegetables (33%). Food self-reliance, incorporating processed foods, was highest for dairy (70%), and vegetables (22%). Dietary self-reliance, comparing local production to dietary recommendations, was less than 20% across food groups. Loss-adjusted availability fell short of dietary recommendations for fruits and vegetables but exceeded recommended levels for grains, oils, and protein foods.

**Discussion:**

These findings highlight critical gaps in local food production and inform strategies to align availability with nutritional goals. This approach and its metrics can be instrumental for other island territories, offering an approach to monitoring self-reliance in non-independent contexts.

## 1 Introduction

Small Island Developing States (SIDS) comprise a geographically dispersed group of 39 countries and 18 territories with shared social, economic, and environmental vulnerabilities (1). These low-lying coastal nations—primarily located in the Caribbean and Pacific—face complex challenges including food insecurity, nutrition-related issues, and the impacts of climate change, such as rising sea levels and natural disasters. In SIDS, the quality, types, and frequency of data are often not sufficient to address the range of questions needed to enable food systems transformation (2, 3). This gap in data availability can be attributed to a lack of available resources, limited local statistical capacity, and varying priorities with regard to food systems (3–6). Food availability and food security research is limited to SIDS countries (2, 7), but studies that cover non-independent territories are scarce. With limited scientific research on non-independent islands, trends in food supply, diets, and food systems risks are understudied and, presumably, undermanaged for over 5.4 million people (8) in vulnerable contexts. To address local food system challenges—be it through policy, management, or innovation—data collection and interpretation at the local level is necessary. While food availability, imports, exports, and production data are available for SIDS countries through the Food and Agriculture Organization Database (FAOSTAT), these data are not available for most non independent territories within the SIDS designation. Inconsistent or sparse agricultural census implementation and hard-to-access trade records complicate food supply assessments of non-independent territories, for which data are not reported by their governing countries (9). In the case of territories of the United States (US), food intake and availability are not represented in broader US data series.

Puerto Rico (PR), an island territory of the US with 3.2 million inhabitants, is hindered by such data gaps, despite pressing needs, making it an ideal candidate for case analysis. Although food insecurity is not monitored in PR through any national or federal surveys, data from a recent study indicate that food insecurity in PR, assessed via the US Household Food Security Survey Module, could be up to 3.8 times higher than in the US (10, 11). In addition to food insecurity, poor nutrition and diet-related chronic disease may be widespread (12, 13), yet very little is known about the local food context, including food insecurity rates, food consumption or availability, and prices. In the US, the National Health and Nutrition Examination Survey (NHANES) includes a dietary intake interview component that provides representative food intake estimates (14), but PR is not included in NHANES; no other representative food intake estimates are available. Similarly, the Food Availability data series provides estimates of the amount of food available for consumption in the US (15). The US Department of Agriculture’s Economic Research Service (USDA ERS) manages and disseminates the Food Availability data series within the Food Availability Data System (FADS) (16). FADS does not include data on the food supply in PR. In PR, the Department of Agriculture (PRDA) reports production and import data for major agricultural products, but these disaggregated records do not describe the entire food supply and are not consistently available (17). In April 2023, the PR Statistics Institute released an online tool to improve accessibility, though it only summarizes data for products with local production (18).

Heavy reliance on food imports can expose SIDS and island territories like PR to global supply chain disruptions, natural disasters, and rising food costs. Prioritizing self-reliance through local food production aligns with efforts to enhance food security, resilience, and sustainability in the face of climate and economic challenges. Self-reliance studies quantify the net balance between production, imports, exports, and consumption of food in a certain geography (19). The concept of food self-reliance is commonly evaluated as a ratio of food demand (consumption) to production and has been evaluated in other regions, such as the Northeastern US (20), and among sovereign countries (21).

In this study, we quantify food availability for PR as a case study, analyzing both the proportion of foods available that originate locally (“agricultural self-reliance” and “food self-reliance”) and the dietary contributions of locally produced foods (“dietary self-reliance”). While islands’ reliance on food imports are commonly cited as a vulnerability, this study aims to assess self-reliance by food group to estimate the gap between local food produced and the necessary amounts to provide a healthy diet to the local population. More broadly, we provide a methodological framework and open-access foundational datasets to support assessment of food availability and self-reliance in other contexts, such as island territories, protectorates, and nations, where such datasets and metrics do not exist and are sorely needed.

## 2 Materials and methods

We evaluate the recent state of food availability in PR in 2016–2019, which includes the effects of Hurricanes Irma and María, recent climatological events which decimated local agricultural production in PR but also avoids the global disruptions in supply chains that occurred during and immediately after lockdowns caused by the COVID-19 pandemic. Here, we briefly summarize our methodological approach, with detail provided in Supplementary material 1.

First, a data crosswalk was constructed to consistently identify traded food items by food groups. Second, using this crosswalk, food availability data for PR for the 2016–2019 period were constructed using publicly available data sources from various government agencies, as described in Table 1. The data used covered local agricultural production, food imports and exports, losses, and waste (Table 1). Third, food availability datasets were used to estimate three self-reliance metrics, including a novel metric of “dietary self-reliance” that compares local production with dietary recommendations. This analysis distinguishes between fresh and processed foods, as defined below. This distinction was made to make a fair comparison between local production and total availability given that local food production data are only available at the farm-gate (fresh).

**Table 1:** Description of data sources used in the development of food availability datasets for Puerto Rico.

Data type	Source (s)	Description	Use
Agricultural production	PR Department of Agriculture (17)	National agricultural production records from farmers in PR. Data collected by the PRDA provide crop, livestock, and fish production information on a yearly basis.	Annual production for each crop produced during the study period was identified using PRDA gross agricultural income reports provided upon request by the Statistics Office of the PRDA.
	PR Institute of Statistics (18)	Online database of local agricultural production.	Used to complement PRDA data when reported units needed to be converted to generalizable units (i.e., millar to quintal or mazos to quintal).
Imports and exports	PR Planning Board (26)	Trade data are collected by the PR Planning Board (PRPB) and are available on a Fiscal Year (FY) basis, Imports represent shipments from the US or foreign countries. Exports represent shipments from PR to the US and foreign countries. PRPB trade data are cataloged by HS commodity codes.	External trade data are available publicly, but this study used workbook versions of the data provided by the PRPB upon the authors’ request. Including the study period (FY 2017, FY 2018, and FY 2019). Over 1,200 different foods, food products and food preparations were estimated to be imported and exported each year, and these products are not disaggregated by commodity composition.
Population data	US Census Bureau (54–56)	Annual demographic estimates are published for PR and its municipalities by the Population Estimates Program of the US Census Bureau through the Puerto Rico Community Survey (PRCS).	Population data were necessary to define per capita dietary requirements for the population living in PR during the study period. Considering that there was a significant decrease in the local PR population after Hurricane María in 2017, population estimates were allocated as follows for the analysis: For FY 2017, the 2016 PRCS 1-year estimates were used; for FY 2018, the 2017 PRCS 1-year estimates were used; for FY 2019 the 2018 PRCS 1-year estimates were used. Population estimates for people 5 years and older were used, as that is the age range that most closely aligns with the Dietary Guidelines for Americans (DGA).
Conversion factors for losses	Loss-Adjusted Food Availability (LAFA) data series produced by USDA’s Economic Research Service (ERS) (27)	Provides estimates of the annual per capita food availability in the US adjusted for losses that occur across the food value chain. LAFA data series provides separate loss estimates for losses that occur from farm to retail, from retail to consumer, and losses at the consumer stage for over 200 commodities.	While definitions of food loss and waste vary around the world, here we retain the terminology used by ERS in which *food-loss* estimates represent the edible amount of food, postharvest, that is available for human consumption but is not consumed for any reason (including cooking loss; natural moisture loss; loss from mold, pests, or inadequate climate control; and food waste) (15).
Food pattern-equivalents	LAFA data series produced by USDA’s ERS (25)	LAFA also provides conversion factors from mass to DGA food pattern-equivalents (FPEQ). FPEQs convert mass-based amounts of foods and beverages in the Food and Nutrient Database for Dietary Studies into food-pattern components.*	Conversion used to express availability in FPEQ were manually added to the HS Food Categorization Crosswalk and applied to estimate food availability in the units used by the DGA in their recommendations.

*Further details are provided in Supplementary material 1.

### 2.1 Harmonized system food categorization crosswalk

Given that most food in PR is imported, we relied heavily on import and export data in which each item is identified with a unique commodity code. The commodity codes are derived from the Harmonized System (HS), which is the global system of nomenclature applied to most world trade in goods (22). To reliably exclude non-food agricultural products from the analysis, and to group food items by food group and degree of processing, we constructed a data crosswalk prior to building the food availability dataset. We developed the HS Food Categorization Crosswalk, which is an Excel-based workbook that merges various datasets to link multiple attributes to food commodities based on their HS codes. This Crosswalk categorizes more than 8,000 different agricultural products. While the focus of this research is on PR, the HS Food Categorization Crosswalk can be directly used or modified for research in other geographies, such as SIDS and is available open access (see Supplementary Dataset 1).

Because one of our aims was to compare local production to dietary recommendations, we mapped commodities to each of the food groups and subgroups included in the 2020–2025 Dietary Guidelines for Americans (DGA) by degree of processing (23). The DGA food groups (and subgroups) included were: vegetables (dark green, red and orange, legumes, starchy, other); fruits; grains; dairy; protein foods (meats, seafood, nuts, seeds, and soy); and oils (from plant-based origin). The degree of processing was either fresh or processed. Foods were categorized as fresh if they were raw, single-ingredient foods that had not undergone any degree of processing beyond harvest and packaging; unmilled grains and foods that are frozen to preserve quality such as frozen fruits, vegetables, and meats were also included in this category (*n* = 2,072). Foods were categorized as processed if they had undergone any degree of processing or included more than one ingredient. Milled grains, such as flours, oils, and processed food products were included in this category (*n* = 1,948). Food items that could not be grouped into one of the DGA food groups were included in the crosswalk but categorized separately to be excluded from subsequent analyses (i.e., coffee, sugar, candy confections, alcoholic beverages; *n* = 1,018). Finally, agricultural items that were not intended to serve as human food were included in the crosswalk but categorized separately to be excluded from the analysis (i.e., grains used for seeds, live animals, animal feed, tobacco products, etc.; *n* = 3,302). There are certain food items, especially grains, which can be used for human food or animal feed. These were not cataloged separately in the crosswalk and were subtracted during the analysis process (as should subsequent studies using the crosswalk analyze human food availability). Of the total HS codes available for agricultural commodities in the Global Agricultural Trade System (GATS), *n* = 4,020 were categorized as into DGA food groups by degree of processing (24) (see Supplementary Dataset 1 and Supplementary material 1).

Each agricultural commodity in the HS that corresponded to a DGA food group was linked to additional data. All levels of losses identified in USDA’s Loss Adjusted Food Availability (LAFA) dataset were documented, when applicable, to food items in the Crosswalk. LAFA conversion factors for mass per Food Pattern Equivalent (FPEQ), the servings unit used by the USDA, were also added to food items in the crosswalk (25) (Supplementary Datasets 1, 2; Supplementary material 1). When LAFA factors were not an exact match for the HS commodity, alternate factors were also identified for the HS food, if available.

### 2.2 Food availability

**Figure 1:**
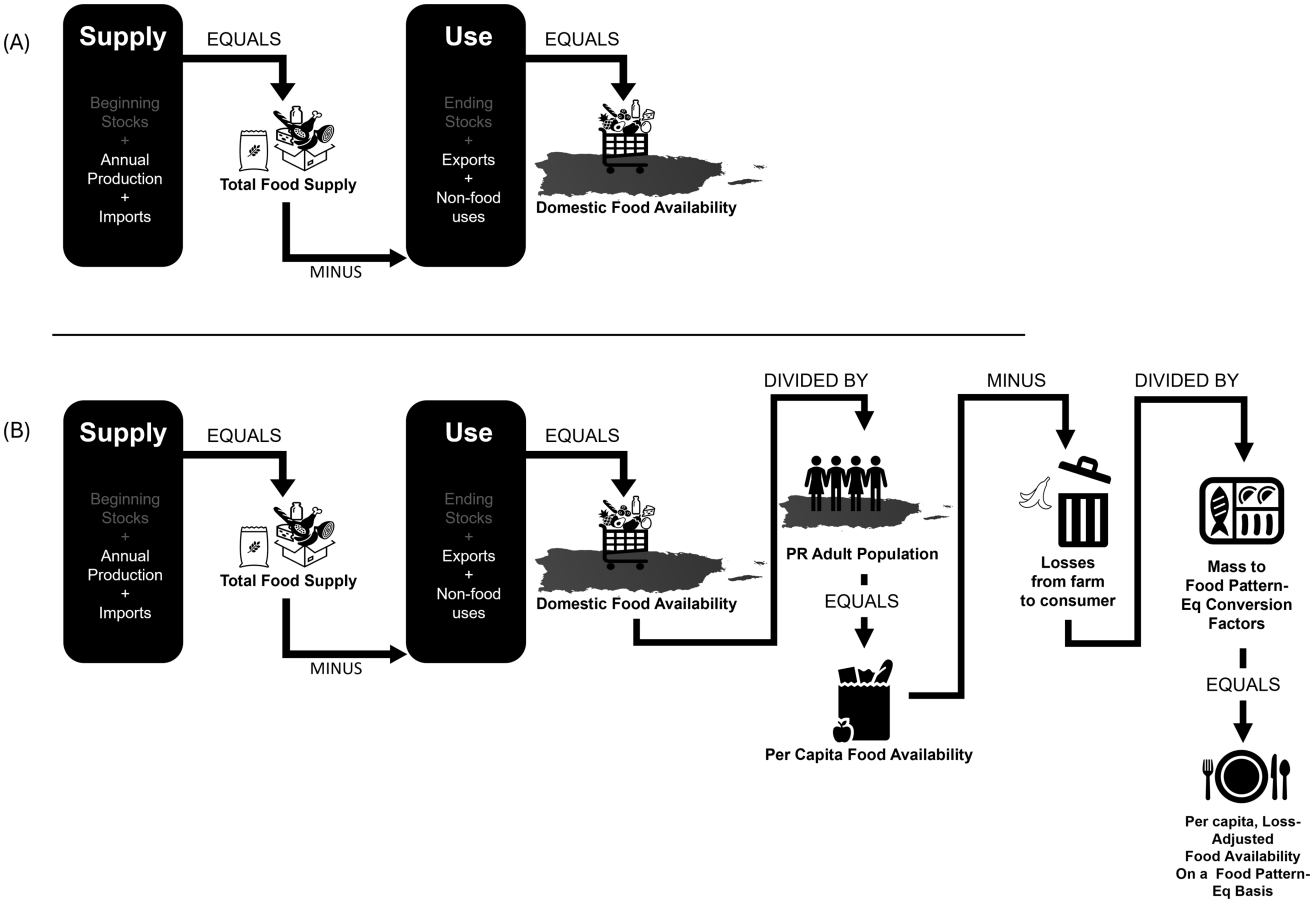
Method for estimating: **(A)** food availability in Puerto Rico and **(B)** per-capita loss-adjusted food availability on a food pattern-equivalent basis. In the Food Availability Data System (FADS) (27), food availability data were computed as the balance between supply (beginning stocks, annual production, and imports) and disappearance (exports, non-food uses and ending stocks) and refined for losses (such as moisture losses, spoilage, and non-edible parts). In this study, the total food supply was estimated from local food production and trade records. There are no public records of local commodity stocks in PR; these estimates were therefore not included. Image is adapted from Figure 1 in “Updated Supermarket Shrink Estimates for Fresh Foods and Their Implications for ERS Loss-Adjusted Food Availability Data” to match the current methodology (15). The term “supply” indicates sources of agricultural products, use’ indicates destination of agricultural products other than human consumption. The net of “supply” and “use” is food available domestically. Icons are free of copyright and obtained from Microsoft 365 library. Puerto Rico Map silhouette is attributed to Natasha Sinegina, published by Creazilla licensed under a Creative Commons Attribution 4.0 license.

We constructed annual food availability datasets for PR by adapting the USDA’s Food Availability Data Series (FADS) method, as summarized in Figure 1A. Food availability was only analyzed for foods that corresponded to the DGA food groups. To estimate food availability, food exports and agricultural products that were suitable for human consumption but were destined for non-food uses, were subtracted from the total food supply (17, 18, 26). Agricultural products not suitable for human consumption were excluded from the analysis. Domestic food availability was estimated by DGA food group and subgroup as the net of supply and use in mass or volume units. Therefore, food availability was estimated for all foods in a certain food group or subgroup (i.e., availability of starchy vegetables) as opposed to the individual level (i.e., availability of potatoes) (see Supplementary material 1). To compare the amounts of food available for people in PR to the DGA, domestic food availability was adjusted to represent the per-capita amount of food available on an as-consumed basis in FPEQ (Figure 1B). Per capita food availability was estimated by dividing domestic food availability by the adult population in PR. Food availability data overstate the actual amount of food eaten by including substantial quantities of food lost from spoilage, moisture loss, and food waste beyond the farm gate in the marketing system (27). Therefore, LAFA loss-adjustment factors were applied to account for losses at the retail and consumer levels.

### 2.3 Self-reliance metrics

To evaluate the current contributions of the local agricultural system to self-reliance, *Agricultural Self-Reliance* (ASR) was estimated as the proportion of local fresh food production and local fresh food availability by food group and year (Equation 1). For ASR, production and availability are adjusted for losses using LAFA loss factors. ASR compares local fresh food production data to fresh food availability for a fair comparison of the additional agricultural production that would be necessary to meet local consumption of fresh foods. Local fresh food production was estimated as the sum of all individual farm-gate food products produced locally that belong to each food group (x) for each year (y), adjusted for losses and in kilograms. Similarly, fresh food availability was estimated as the sum of all available fresh food products that belong to each food group (x) for each year (y), adjusted for losses and in kilograms.


(1) $$\text{Agricultural Self Relianc}e_{x,y}=\frac{\begin{array}{c} \text{Loss adjusted local fresh food}\;\\ \text{productio}n_{x,y} \end{array}}{\begin{array}{c} \text{Loss adjusted fresh food}\;\\ \text{availabilit}y_{x,y} \end{array}}$$

We define *Food Self-Reliance* (FSR) as the proportion of local food production that contributes to total food availability by food group (Equation 2). FSR is the positive opposite of import dependence. The numerators of FSR and ASR are the same, but the denominator of FSR includes both fresh and processed foods (total food availability). Total food availability was estimated as the sum of all available fresh and processed food products that belong to each food group (x) for each year (y), adjusted for losses and in kilograms.


(2) $$\text{Food Self Relianc}e_{x,y}=\frac{\begin{array}{c} \text{Loss adjusted local fresh food}\;\\ \text{productio}n_{x,y} \end{array}}{\begin{array}{c} \text{Loss adjusted fresh and processed}\;\\ \text{food availabilit}y_{x,y} \end{array}}$$

To compare food availability to DGA recommendations, self-reliance was estimated based on dietary recommendations by food group and described as *Dietary Self-Reliance* (DSR). Recommended daily intake for each food group was based on a 2,000-calorie diet and the “Healthy US-Style Dietary Pattern” in the 2020–2025 DGA. DSR was estimated as shown in Equation 3, where loss-adjusted local fresh food production is calculated as in Equation 1 but converted and divided by total population to derive per capita estimates in FPEQ for each food group (x) and year (y) and per capita recommended intake for each food group (x) and year (y) is derived according to the DGA.


(3) $$\text{Dietary Self Relianc}e_{x,y}=\frac{\begin{array}{c} \;\mathrm{Per}\;\text{capita loss adjusted local fresh}\;\\ \text{food productio}n_{x,y} \end{array}}{\mathrm{Per}\;\text{capita recommended intak}e_{x,y}}$$

## 3 Results

### 3.1 Food availability

Food availability in PR was assessed for FY 2017, FY 2018, and FY 2019 (hereafter simply referred to as 2017, 2018, and 2019, respectively) (see Supplementary material 2 for individual year results). Annual average results are presented in Table 2. Food availability is presented as the net of local production, imports, and exports adjusted for losses at retail and consumer levels. Some imported foods were re-exported from PR, therefore there were exports reported for food groups for which there is no local production (e.g., oils).

**Table 2:** Summary of foods available in Puerto Rico, annual average FY 2017–FY 2019 in metric tons (MT).

Food group	Local production (MT)	Imports (MT)		Exports (MT)		Losses (MT)		Loss-adjusted food availability (MT)	
	Fresh	Fresh	Processed	Fresh	Processed	Fresh	Processed	Fresh	Processed
Vegetables	**104,863**	**195,714**	**134,387**	**4,998**	**33,261**	**134,330**	**20,629**	**161,249**	**80,497**
Dark green vegetables	14	1,166	0	0	0	440	0	739	0
Red and orange vegetables	23,729	33,942	36,650	1,362	12,552	27,540	6,831	28,769	17,268
Beans, Peas, Lentils^a^	185	3,177	23,751	569	20,253	1,287	21	1,506	3,477
Starchy vegetables	72,592	74,213	44,847	2,815	100	61,755	8,296	82,235	36,451
Other vegetables	8,343	83,216	29,139	253	356	43,307	5,481	47,999	23,302
Fruits	**50,403**	**63,866**	**153,329**	**13,031**	**5,454**	**56,597**	**23,276**	**44,641**	**124,599**
Grains	**15**	**468,729**	**139,128**	**32,848**	**20,554**	**143,077**	**32,832**	**292,819**	**85,741**
Dairy	**225,434**	**14,003**	**106,175**	**2,097**	**10,547**	**70,252**	**35,732**	**167,087**	**59,896**
Protein foods	**41,667**	**288,443**	**94,567**	**1,816**	**3,204**	**118,095**	**23,844**	**210,199**	**67,519**
Meats, poultry, eggs	40,705	273,191	74,746	944	2,989	111,563	19,345	201,389	52,412
Seafood	907	13,423	16,749	851	203	6,110	4,032	7,369	12,515
Nuts, seeds, and soy products	56	1,829	3,071	21	12	422	468	1,441	2,591
Oils^b^	**–**	**–**	**69,155**	**–**	**716**	**–**	**23,185**	**–**	**45,254**

^a^Dried legumes are included among processed foods as they are primarily imported for canning locally. ^b^Does not include fats from animal sources. Bolded values represent food group totals.

On average during the study period, grains, protein foods, and fruits were the most abundant food groups available in PR on a loss-adjusted mass basis (Table 2). The percentage of total food available that was fresh (vs. processed) was higher for green vegetables (100%), meat, poultry, and eggs (79%), grain (77%), dairy (74%), starchy vegetables (69%), other vegetables (67%), and red and orange vegetables (62%) on a loss-adjusted mass basis. Compared to fresh foods, processed foods were most abundant for oils (100%), leguminous vegetables (70%), nuts, seeds, and soy products (64%) and seafood (63%).

Methodological similarities between the LAFA dataset and the Food Availability Datasets produced in this study allow for comparisons between US and PR food availability by food group. To contextualize our food availability results, we provide comparisons with food availability estimates from the US. Table 3 summarizes the results for per capita loss-adjusted food availability data from the LAFA dataset and our results for PR. For vegetables and fruits, per-capita food availability was similar in PR and US. Among food subgroups, however, there were notable differences in availability, particularly for vegetables. For example, while total vegetable availability was only 6% higher in the US than PR, dark green vegetables were 27.2 times more available in the US than in PR. Red and orange vegetables and leguminous vegetables were 71% more available in the US than in PR. In contrast, availability of starchy and other vegetables was 27 and 19% lower in the US than in PR, respectively. While availability of fruits was comparable in PR and in the US, the proportion of fresh fruits available was higher in the US (63% of total fruit availability as fresh vs. 38% in PR).

**Table 3:** Food Availability in the United States and Puerto Rico, on a food pattern-equivalent basis, compared to dietary recommendations, average for FY2017–FY2019.*,**

Food group	Average recommended amount per capita^a^	United States loss-adjusted per capita food availability^b^			Puerto Rico loss-adjusted per capita food availability			
	(FPEQs)	(FPEQs)			(FPEQs)			
		Fresh	Processed	Total	Fresh (imported)	Fresh (local production)	Processed (Imported)	Total^c^
Vegetables (cup eq/day)	**2.5**	1.16	0.72	**1.89**	0.70	0.33	0.75	**1.78**
Dark green vegetables (cup eq/week)	1.5	1.23	–	1.23	0.04	0.00	–	0.05
Red and orange vegetables (cup eq/week)	5.5	1.65	0.97	2.62	0.66	0.45	0.43	1.53
Beans, Peas, Lentils (cup eq/week)	1.5	0.14	1.92	2.06	0.06	0.01	1.25	1.32
Starchy vegetables (cup eq/week)	5	2.54	1.90	4.44	2.01	1.70	2.39	6.10
Other vegetables (cup eq/week)	4	2.57	0.27	2.4	2.15	0.19	1.25	3.52
Fruits (cup eq/day)	**2**	0.52	0.28	**0.80**	0.16	0.12	0.51	**0.79**
Grains (ounce eq/day)	**6**	0.68	6.49	**7.16**	11.65	0.00	2.91	**14.56**
Dairy (cup eq/day)	**3**	0.46	1.03	**1.49**	0.03	0.56	0.73	**1.32**
Protein Foods (ounce eq/day)	**5.5**	6.55	0.13	**6.68**	5.68	0.62	2.03	**8.33**
Meats, poultry, eggs (ounce eq/week)^d^	26	45.84	–	45.84	37.81	4.22	10.44	52.47
Seafood (ounce eq/week)^e^	8	2.04	0.92	2.96	1.47	0.11	2.70	4.28
Nuts, seeds, and soy products (ounce eq/week)^f^	5	7.08	–	7.08	0.60	0.02	1.10	1.72
Oils (grams/day)^g^	**27**	–	44.70	**44.70**	**–**	–	36.94	**36.94**

*Data for Puerto Rico represent FY 2017–FY 2019 while data for United States Represent calendar years 2017–2019. **Population estimates for Puerto Rico are from the American Community Survey.

a Dietary recommendations are based on 2,000-calorie diet following a “Healthy US-Style Dietary Pattern” described by the DGA.

b Weekly availability was estimated by from daily availability from LAFA times 7.

c Total = Fresh Imported + Fresh Local Production + Processed Imported; deviations are due to rounding.

d LAFA data are only available at the un-processed level for meats.

e LAFA data for seafood are only available through 2018.

f LAFA dataset does not include soy products. Processed nut products are not differentiated. LAFA data for peanuts, tree nuts, and coconuts is available through 2018.

g Availability of oils in the US is based on LAFA data for “salad and cooking oils” which is available through 2010. Availability of oils in PR is based on oils from vegetable sources. Bolded values represent food group totals.

Per-capita availability of protein foods, oils, and grains was substantially different between the US and PR. Protein foods were 24% more available in PR, with variations across subgroups. Seafood availability was 45% higher in PR, and the availability of meat, poultry and eggs was 14% higher in PR compared to the US. For plant-based proteins, however, availability in the US was over 4 times higher compared to PR. Oils are 21% more abundant on a per-capita basis in the US than in PR. Total per capita grain availability is 51% lower in the US than in PR, though grain availability is likely overestimated, as an unknown quantity is destined for animal feed (see Limitations).

### 3.2 Agricultural self-reliance

**Figure 2:**
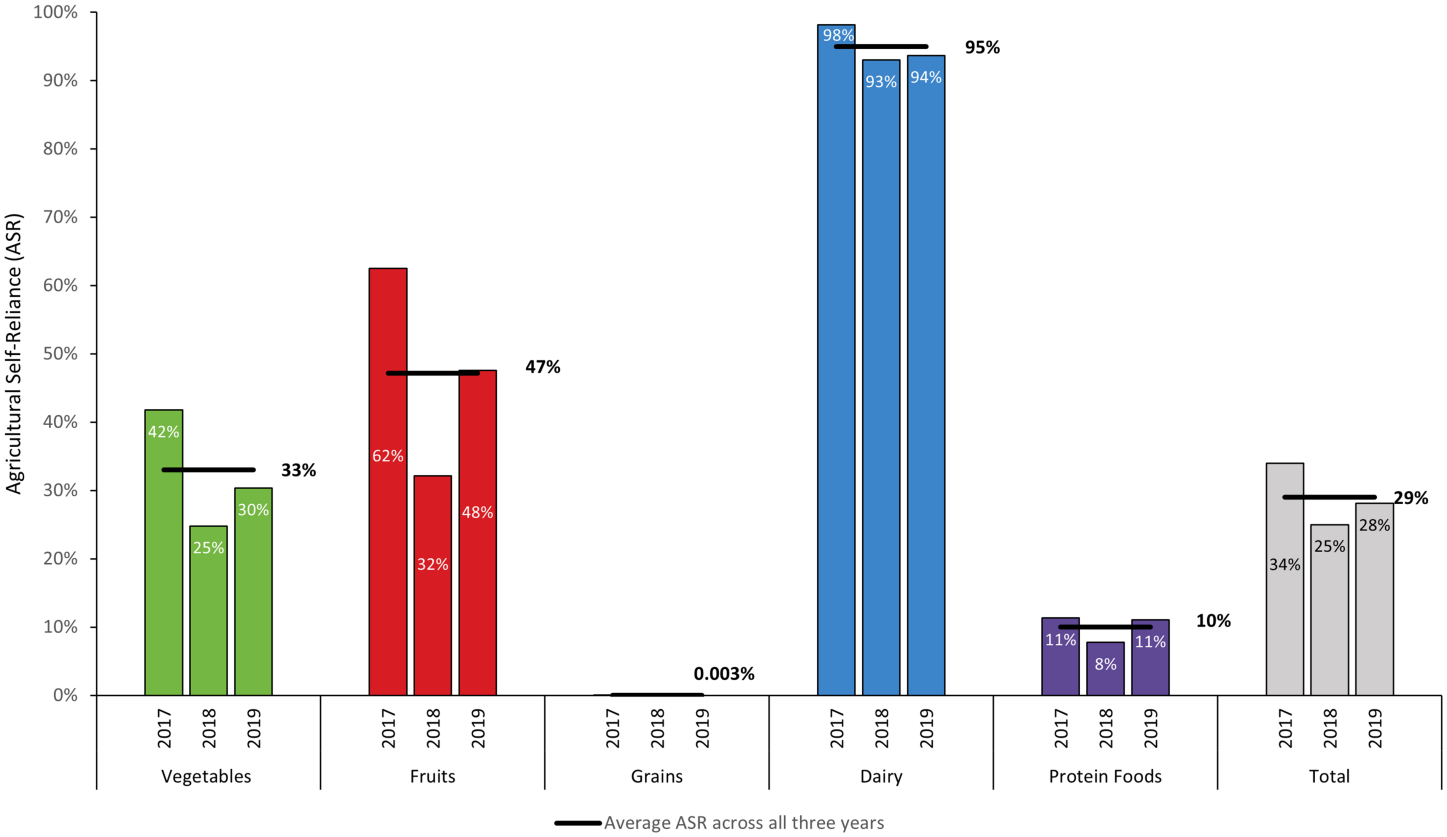
Agricultural self-reliance (ASR) in Puerto Rico by food group: proportion of fresh foods available provided by domestic production adjusted for losses on a mass basis (FY 2017–FY 2019).

ASR results show the proportional contribution of locally produced fresh foods to total fresh food availability by food group, adjusted for losses. Total ASR was higher in 2017 than in subsequent years (34%) and lowest in 2018 (25%) (Figure 2). In 2019, total ASR increased relative to 2018, but did not return to 2017 levels. On average during the study period, ASR was highest for dairy (95%), followed by fruits (47%) and vegetables (33%). ASR was 0.003% for grains. ASR for oils was not calculated as oils were not classified as fresh products.

For the dairy group, local dairy production consisted of fresh cow’s milk. Dairy ASR remained high throughout the study period, with only a 5.3% decrease and 4.6% decrease relative to 2017 in subsequent years, respectively. ASR for fresh fruits decreased throughout the study period. Relative to 2017, ASR for fresh fruits decreased by 49% in 2018 and by 24% in 2019. For fresh vegetables, ASR decreased throughout the study period, with ASR 41% lower in 2018 and 27% lower in 2019 relative to 2017 (see Supplementary material 2). On average, local production was highest for starchy vegetables (69% of total fresh vegetable production), red and orange vegetables (23% of total), and other vegetables (8% of total) throughout the study period. Meat, poultry, and eggs contributed to the majority of locally produced fresh protein foods (97%) while seafood contributed 2% of local fresh protein foods. For the nuts, seeds, and soy production category of protein foods, local production consisted of fresh coconuts and contributed 0.2% of total protein food production (Table 2). As with vegetables, ASR for protein foods declined substantially in 2018 (32% lower than 2017). However, unlike vegetables, by 2019 ASR for protein foods rebounded to be only 2.5% lower than 2017.

### 3.3 Food self-reliance

**Figure 3:**
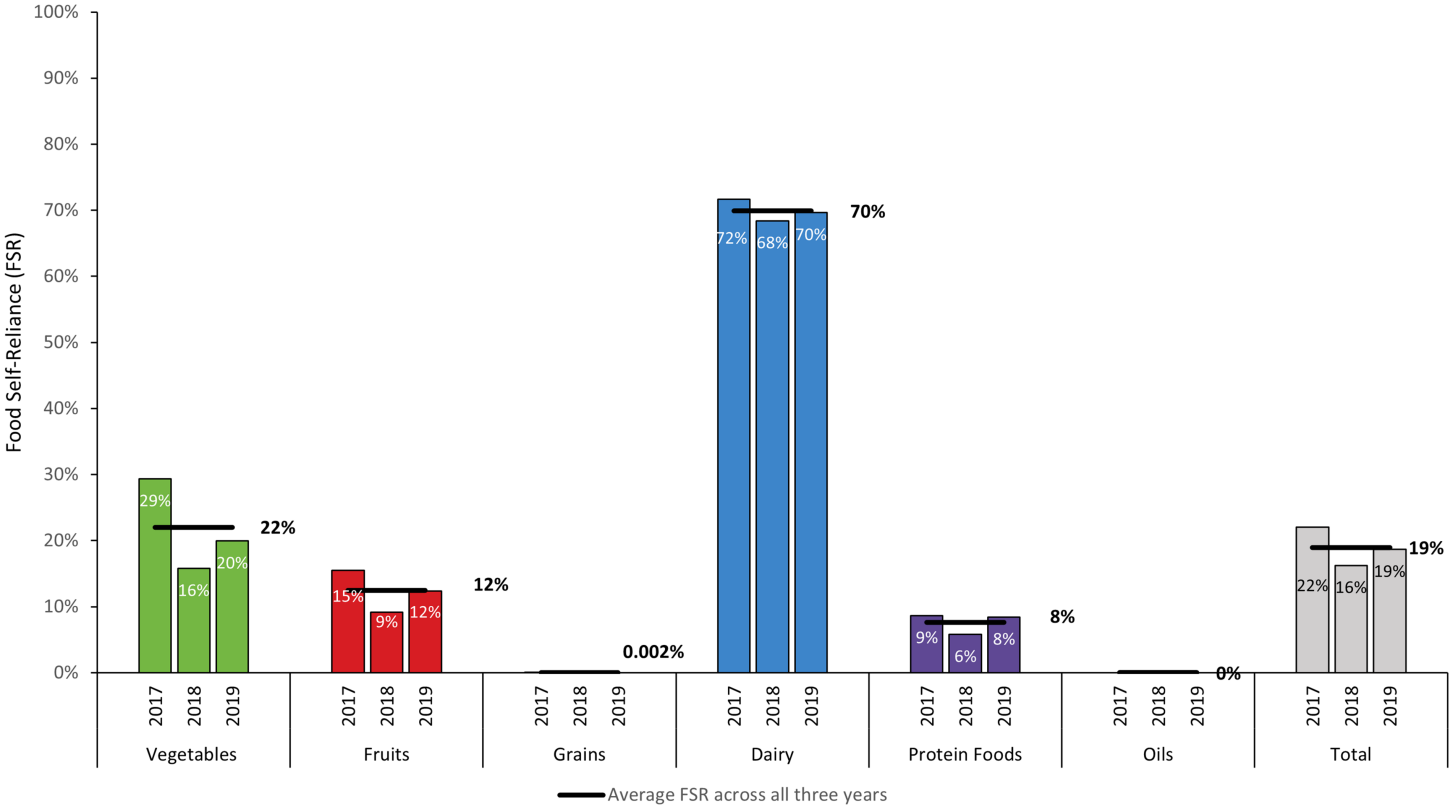
Food self-reliance (FSR) in Puerto Rico by food group: proportion of total (fresh and processed) foods available provided by domestic production adjusted for losses on a mass basis (FY 2017–FY 2019).

Total FSR was higher in 2017 than in subsequent years (22%) and lowest in 2018 (16%) (Figure 3). In 2019, total FSR increased relative to 2018, but did not return to 2017 levels. Total FSR and FSR for individual food groups behaved like ASR over the study period. Considering fresh and processed foods available, adjusted for losses, the proportion of those foods supplied by local agricultural production (food self-reliance) was lowest for oils (0.0%), grains (0.002%), protein foods (8%), fruits (12%), and vegetables (22%) on average over the study period. Food self-reliance was highest for dairy (70%). Food self-reliance across all food groups averaged 19% throughout the study period (Figure 3).

Dairy FSR remained high throughout the study period, with only a 5% decrease and 3% decrease relative to 2017 in subsequent years, respectively. FSR for fruits decreased throughout the study period. Relative to 2017, FSR for fresh fruits decreased by 41% in 2018 and by 20% in 2019. For vegetables, FSR decreased throughout the study period, with FSR 46% lower in 2018 and 32% lower in 2019 relative to 2017 (see Supplementary material 2). As with vegetables, FSR for protein foods declined substantially in 2018 (33% lower than 2017). However, unlike vegetables, by 2019 ASR for protein foods rebounded to be only 2.6% lower than 2017.

### 3.4 Dietary self-reliance

**Figure 4:**
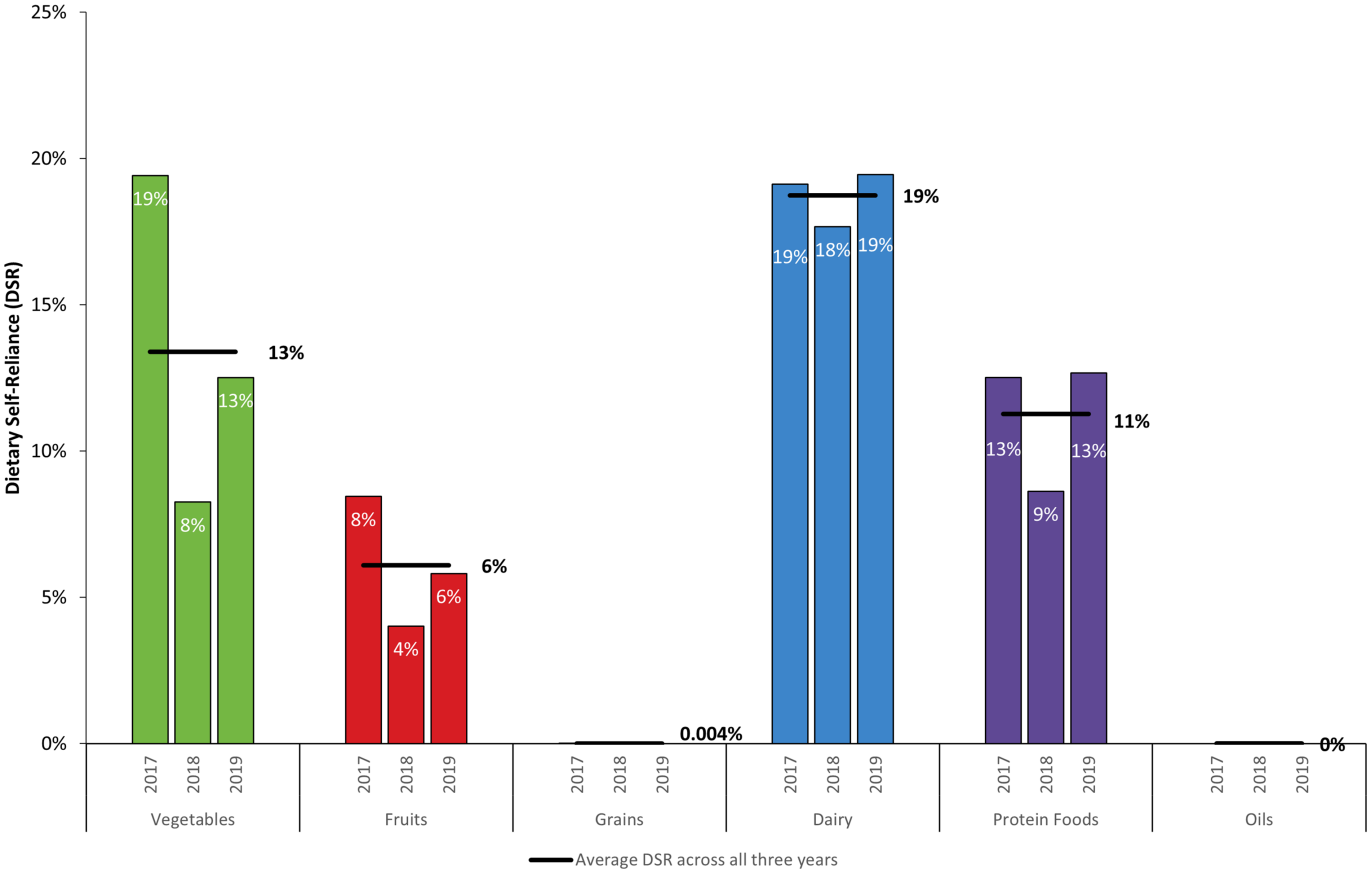
Dietary self-reliance (DSR) by DGA food group on a food pattern-equivalent basis (FY 2017–FY 2019). Dietary recommendations are based on 2,000-calorie diet following a “Healthy US-Style Dietary Pattern” described by the DGA.

DSR is the proportion of Dietary Guidelines for Americans (DGA) recommendations met by locally produced fresh foods in PR. A total DSR was not estimated because recommended amounts are specified in different unit-equivalents across food groups (i.e., cup-equivalents for fruits and vegetables and ounce-equivalents for proteins). Compared to the DGA recommendations, an average of 19% of the recommended dairy intake was provided by local dairy production during the study period (Figure 4). On average, DSR was 13% for vegetables, 11% for protein foods, and 6% for fruits. Average DSR for grains was 0.004 and 0% for oils during the study period.

DSR declined across all food groups in 2018 relative to 2017, although reductions were higher for vegetables and fruits. In 2019, DSR for dairy and protein foods rebounded, but remained lower than in 2017 for vegetables and fruits. DSR decreased by 57% for vegetables in 2018 and by 36% in 2019 relative to 2017. Among vegetable subgroups, DSR for starchy vegetables was highest (34% DSR during the study period, see Table 4 and Supplementary material 2). DSR for starchy vegetables decreased from 52% in 2017 to 15% in 2018 and 35% in 2019. DSR for the other vegetables subgroup was 5% on average during the study period. In 2018 and 2019, DSR was 33% lower than in 2017 for other vegetables. During the study period, 8% of the recommended intake for red and orange vegetables came from local production. Relative to 2017, DSR for red and orange vegetables decreased by 10% in 2018 and by 50% in 2019. DSR values for the legumes and green vegetables subgroup were less than 1% during the study period at 0.4 and 0.05%, respectively. DSR for fruits was 6% on average during the study period. For fruits, DSR decreased from 8% in 2017 to 4% (52% lower) in 2018 and was 5% in 2019 (31% lower than in 2017).

**Table 4:** Mean agricultural self-reliance (ASR), food self-reliance (FSR), and dietary self-reliance (DSR) for Puerto Rico, average for FY 2017–FY 2019.

Food group category	Mean ASR	Mean FSR	Mean DSR
	(%)	(%)	(%)
Vegetables	**33.0**	**22.0**	**13.4**
Dark green vegetables	1.0	1.0	0.05
Red and orange vegetables	43.5	27.2	8.2
Beans, Peas, Lentils	6.5	2.0	0.4
Starchy vegetables	44.5	30.8	34.0
Other vegetables	8.5	5.7	4.8
Fruits	**47.2**	**12.4**	**6.1**
Grains	**0.003**	**0.002**	**0.004**
Dairy	**95.0**	**69.9**	**18.7**
Protein foods	**10.0**	**7.6**	**11.3**
Meats, poultry, eggs	10.2	8.1	16.2
Seafood	6.7	2.5	1.3
Nuts, seeds, and soy products	3.3	1.2	0.4
Oils	**0.0**	**0.0**	**0.0**
All food groups	**29.0**	**19.0**	**–**

Bolded values represent food group totals.

The per-capita proportion of available dairy foods provided by local production remained stable throughout the study period (19% average DSR) with an 8% decline in DSR in 2018 but a 2% increase in 2019 relative to 2017. While the DSR for protein foods was 11% throughout the study period, DSR for the meats, poultry, and eggs subgroup was 16% during this period. For seafood, DSR was 1 and 0.4% for nuts, seeds, and soy products. DSR for meats, poultry and eggs decreased by 32% in 2018, from 18 to 12% DSR, and increased by 2% in 2019 relative to 2017 reaching 18.4% DSR. For seafood, DSR was 1.2% in 2017, and it increased by 35% in 2018 reaching 1.6% DSR. In 2019 DSR for seafood returned to the 2017 level at 1.2%. Given that DSR considers population size, shifts in DSR throughout the study period could be associated with the impacts of climatological events in 2018 as well as the related population decrease that happened afterwards.

### 3.5 Food availability, dietary recommendations, and self-reliance metrics

**Figure 5:**
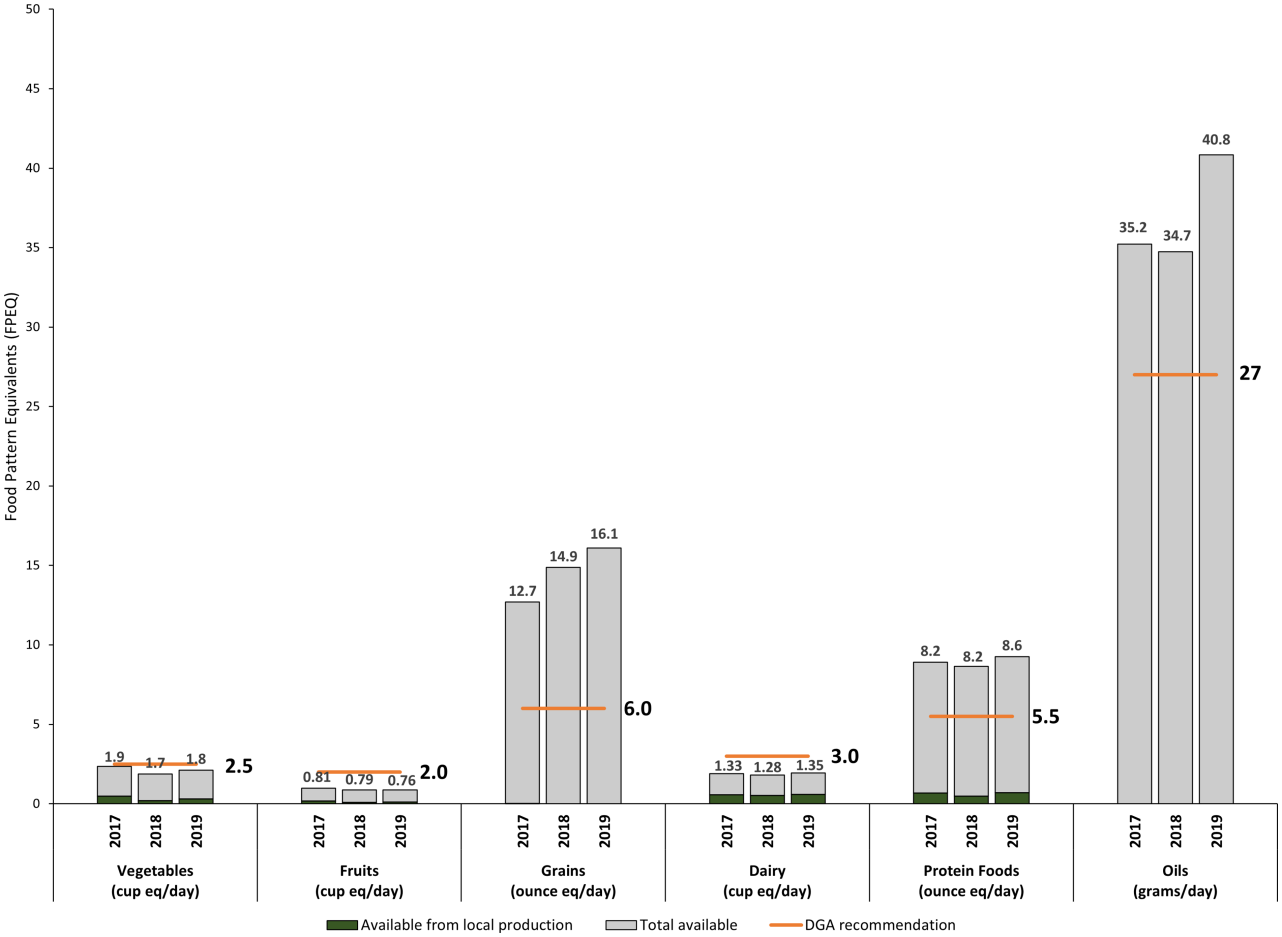
Loss-adjusted per-capita availability of food in Puerto Rico, identifying local production, compared to DGA recommendations by food group on a food pattern equivalent basis (FY 2017–FY 2019).

By comparing dietary recommendations against total food availability, it is possible to evaluate whether the amount and types of food available in PR’s food supply are aligned with the healthy eating patterns recommended by the US government (Figure 5).

On average, loss-adjusted per capita food availability exceeded DGA recommendations for several food groups, including grains (243% or 2.4 times the recommended amount), oils (137% or 1.4 times the recommended amount), and protein foods (151% or 1.5 times the recommended amount) (Figure 5). For protein foods, local protein production contributed 10% of all available fresh protein foods (ASR), 8% of all available protein foods (FSR), and 11% of the dietary recommendations for protein foods (DSR) (Table 4). Considering all sources of food, availability of protein food exceeds the recommended per capita amounts (Table 3).

Loss-adjusted per capita food availability was lower than DGA recommendations for vegetables (0.71 times the recommended amount), fruits (0.39 times the recommended amount), and dairy (0.44 times the recommended amount) (Table 3). While 95% of fresh dairy available was locally produced (ASR), local production only satisfied 19% of dietary recommendations (DSR) (Table 4). Regardless of origin or degree of processing, locally available dairy was less than required to meet the DGA recommendations among adults living in PR (Figure 5, Table 3, and Supplementary material 2). Similarly, for vegetables, almost 1/3 of fresh vegetable availability was supplied locally (ASR: 32%), but this amounts to meeting only 13% of the DGA recommendation (DSR; Table 4). Local fresh fruit production contributed to 47% of the total fresh fruit available locally (ASR), but only 6% of the dietary recommendations (DSR) (Table 4).

Results for ASR and FSR are comparable for oils (0% ASR and 0% FSR), grains (0.003 and 0.002%), and proteins (10 and 8%) for which processed foods accounted for nearly 1/4 of the total available. ASR was higher than FSR for vegetables (33% ASR and 22% FSR) with nearly 1/3 of total vegetables available in processed form. The highest difference between ASR and FSR was for fruits (47 and 12%) indicating that a higher proportion (nearly 3/4) of these foods were available in processed form. ASR and FSR were highest for dairy (95% ASR and 70% FSR) with 1/4 of all available dairy being in processed form. During the study period, ASR was 29% on average, while FSR 19% (Table 4).

FSR and ASR are different for most food groups (except oils). This discrepancy is because FSR includes all fresh and processed foods in the denominator while ASR only includes fresh foods. ASR compares local agricultural production with total availability of fresh foods and therefore offers insight into the proportion of available fresh food that could originate from local agricultural production independent of processing infrastructure. By considering processed foods, significant changes to FSR would require increased agricultural production as well as food processing locally. Evaluating the current contributions of local food processing is beyond the scope of the study at hand (see Limitations). The contrasting estimates offered by FSR and ASR values suggest that planning for increased self-reliance should consider the types of foods that are being substituted by local production. The availability of food processing infrastructure and the overall viability of such developments would influence the trajectory toward increased self-reliance, while agricultural potential and farmland accessibility would be a more definitive factor influencing ASR.

## 4 Discussion

We developed a publicly available food availability dataset for PR, quantifying the extent to which local food production meets food demand and dietary recommendations. This work is the first comprehensive analysis of contemporary food availability using trade and local production records, providing a foundation for future monitoring and evaluation of PR’s food system. PR’s food system shares many characteristics with SIDS, including reliance on imports, climate vulnerabilities, and limited infrastructure. Our study methodology is broadly applicable to non-independent SIDS territories, providing a framework to assess food systems when production and trade data are disaggregated. Our approach leverages widely used HS codes to merge datasets.

For PR specifically, food balance sheets in 1973 indicated heavy reliance on imported food with 100% of available cereals, 99% of fats and oils, and 59% of vegetables being imported (28). Our study shows that this trend has persisted and, in some cases, increased (Table 2). For example, during the study period, 74% of vegetables, 90% of proteins, and 80% of fruits were imported. Since 1973, land in agricultural production has declined by 63% (29, 30) and population has increased, reflecting shifts in technologies, global food systems, and consumer preferences. While local milk production remains relatively robust, focusing primarily on fluid milk, most dairy imports consist of processed products.

Previous studies, such as Comas-Pagán, estimated that 80% of PR’s food supply was imported, with the majority comprising cereals, oils, and legumes (31). Our findings align broadly but suggest slightly higher import dependence (81%, derived from FSR of 19%; see Table 4) due to continued declines in agricultural land, the impact of hurricanes, and differences in methodology. Notably, our inclusion of processed foods highlights a broader spectrum of import reliance compared to prior analyses.

Our study advances the conversation on food import substitution by estimating the proportion of available fresh foods provided by local production (ASR). While total FSR was 19% (Figure 3), fresh food ASR was higher at 29%, emphasizing the potential to reduce import dependence by bolstering local fresh food production. Import dependence has fostered a diabetogenic food environment composed of processed and non-traditional foods across SIDS (32). Promoting fresh food consumption could improve diet quality at lower infrastructure investment costs in SIDS countries and territories. However, achieving this requires context-specific studies, as well as investments in local markets, equitable food access, and consumer acceptance of fresh foods.

The effects of Hurricanes Irma and María in 2017 significantly disrupted agriculture, reducing self-reliance metrics in PR. These hurricanes destroyed up to 80% of crops (33), with the greatest impact in the present study for vegetables and fruits. Among locally grown starchy crops, root crops demonstrated higher resilience than perennial crops (i.e., breadfruit, plantain). Root crops have shorter growing cycles (34); some sprout again easily after foliage loss (i.e., cassava) (35); some tolerate flooded soils (i.e., taro) (36); some are resistant to hurricane winds given their vining growth (37); and they require minimal infrastructure (i.e., trellises, staking, refrigerated storage), partly explaining their relative performance. Protein foods and dairy also proved robust during this period, although poultry production suffered disproportionately due to infrastructural losses, lack of insurance coverage, and outmigration (33, 38). While climate change vulnerabilities have been studied across SIDS (39), the unique and detrimental sociopolitical conditions of non-independent island territories are not contemplated in these assessments (40). Monitoring food availability and self-reliance across non-independent SIDS can inform agricultural planning strategies that are adequate for each unique climatological, economic, and socio-political context.

Dietary adequacy remains a challenge in PR (41), as has been documented across SIDS (2, 42). A study evaluating availability of food groups at a SIDS-wide level found that 94% of SIDS fail to meet food group requirements (2). This case study in PR found availability was less than dietary recommendations for vegetables, fruits, and dairy which are all associated with the “adequacy” components of the Healthy Eating Index (HEI) (43), the most commonly used measure of dietary quality based on the DGAs. In contrast, availability was greater than recommendations for grains, protein foods, and oils which, depending on degree of processing, are associated with the “moderation” components of HEI (43). Our results align with prior studies identifying low fruits and vegetable intake (12, 44–46), likely influenced by value chains constrains and extended shipping times for imports (31, 47). A recent study found that independent countries in the Caribbean Community (CARICOM) were only 34% self-sufficient for vegetables, but 149% self-sufficient for fruits (21).

A focused strategy to substitute imports with locally produced vegetables—particularly green, red, and orange vegetables—could enhance diet quality and diversify the local food environment. Investments in crop research, food safety, aggregation, and local procurement activities would support this shift. However, food availability alone does not equate to consumption; cultural preferences and structural barriers must also be addressed. For example, non-starchy vegetables (i.e., leafy greens or cruciferous vegetables), have not been significant part of PR’s gastronomic, economic, or agronomic systems (48, 49). Anthropologist Sydney Mintz mapped how Puerto Rican diets became structured by class, race, and identity (50). As he noted from his observations in the late 1940s, urbanization and dependency on imports marginalized home-grown vegetables in favor of convenient, globally distributed staples (50). A recent study of dietary acculturation among Puerto Rican adults living in the US found that stronger psychological US orientation was associated with higher diet quality, particularly with higher income (51). More Spanish use, stronger psychological Puerto Rican orientation, and shorter length of mainland-US residency were associated with traditional dietary patterns, which are of lower diet quality (51). It is relevant to note that PR’s GDP per capita was $36,779 in 2023 (52) while it was projected at $7,094 for 2024 in the Caribbean SIDS (53). This non-trivial difference highlights the need for country-specific data and analyses across SIDS, especially non-independent territories, as solutions to food import dependence must be contextualized.

This study contributes to a growing body of literature on food systems in SIDS, offering actionable insights for PR and comparable contexts. By quantifying food availability and self-reliance, we provide a foundation for evaluating the healthfulness and resilience of local food systems. Future research should explore nutrient availability, food preferences, and strategies to strengthen local food production, particularly in the face of climate change, including addressing issues of access to agricultural lands, land tenure, and youth agricultural education programs.

### 4.1 Limitations

This study models PR’s local food supply by simplifying trade transactions and applies conversion factors based on US data, which introduces several limitations. It relies on PRDA data (17, 18), representing only a subset of farmers in PR, likely underrepresenting local food production and underestimating self-reliance. Our self-reliance results are therefore conservative, though the magnitude of potential underestimation is unknown. Assumptions about food form and processing were made due to a lack of data on local food processing activities. U. S.-based loss estimates were applied, potentially underestimating losses in PR, especially for fresh foods, given extended shipping times for imports. Additionally, dent corn #2 and #3, destined for animal feed, were excluded due to insufficient data from local grain mills. Given that local food production data are only available for fresh agricultural products, the results of this analysis can only be interpreted in relation to agricultural activities and are not inclusive of further local processing of these agricultural products. Similarly, imported fresh products may be processed locally before being available for consumption and this analysis only considers those foods in their imported form.

## 5 Conclusion and recommendations

This study provides evidence of food self-reliance—the opposite of import dependence—for PR by food group, data that had not been previously made available. To assess self-reliance, a food availability dataset was built from disaggregated records, a data analysis challenge that is true of other non-independent SIDS. By using territory-specific production and trade records, this study offers insights into local food security initiatives. By means of a data crosswalk, the approach used in this study can support similar assessments for other non-independent island territories. Providing coverage of non-independent SIDS by international agencies in existing databases and supporting country-specific loss estimates is imperative to advance these efforts.

The findings of this study underscore the variability of food import dependence across different food groups and highlight the inadequacy of local agricultural production in meeting dietary recommendations during the specified study period in PR. There is a gap between the quantity of locally cultivated food and the recommended food intakes for the local adult population based on DGA recommendations. A food self-reliance strategy for PR must move beyond a singular focus on increasing food sufficiency to ensuring the nutritional adequacy of the food supply to promote health. Data from this study highlight the opportunity to increase local production of vegetables, especially dark green vegetables, and protein foods such as seafood and meats. The prevalence of poverty and food insecurity, coupled with the effects of climate change affecting local food stability, call for substantial policy interventions to address food security comprehensively.

To adequately formulate policies across non-independent SIDS that address food import dependence and food security, representative and longitudinal data are necessary. Efforts should be promptly directed toward incorporating data collection practices into the policy planning process. Implementing robust data collection practices that accurately depict local agricultural production, food intake, local food prices, and food insecurity rates is critical. These data not only facilitate informed policy development at the local level but also hold significance at the federal level, in the case of US territories, emphasizing the broader implications for comprehensive food security initiatives.

## Data Availability

All input data are available through the sources listed in the references. Additional methodological details and year-by-year results can be found in online repositories. The names of the repository/repositories and accession number(s) can be found in the article/Supplementary material.

## Supplementary material

The following Supplementary materials are available through open access via OSF.io (https://doi.org/10.17605/OSF.IO/72VJQ).

Supplementary Dataset 1 HS Food Categorization Crosswalk. The HS Food Categorization Crosswalk is an Excel workbook that merges multiple data sources to assign a food group category, degree of processing, loss factors, and mass per food pattern-equivalent to food items in the Harmonized Trade System.

Supplementary Dataset 2 Summary of estimates used in the analysis. This supplemental dataset summarizes all LAFA loss adjustment factors and food pattern equivalent conversion factors for all food types in the HS Food Categorization Crosswalk. The table summarizes, separately, alternative values for both losses and food pattern equivalencies. In addition, for the comparison between LAFA and PR Food Availability results, this supplement includes the LAFA values used in estimating weekly and daily FPEQ availability.

Supplementary material 1 Data sources, assumptions, and structure of the HS food categorization crosswalk. This supplemental material includes additional details on the construction of the HS Food Categorization Crosswalk as well as the assumptions made during the process of allocating loss factors and mass per food pattern equivalent conversion factors.

Supplementary material 2 Additional tables and figures. This supplemental material includes additional results detailed by year, food group, and subgroups.

## References

[1] United Nations. About small island developing states | office of the high representative for the least developed countries, landlocked developing countries and small island developing states. (2024). Available online at: https://www.un.org/ohrlls/content/about-small-island-developing-states (Accessed February 27, 2025).

[2] Atzori D Sonneveld BGJS Alfarra A Merbis MD. Nutrition fragility in isolation: food insecurity in Small Island developing states. Food Secur. (2024) 16:437–53. doi: 10.1007/s12571-024-01438-z

[3] Marshall Q Bellows AL McLaren R Jones AD Fanzo J. You say you want a data revolution? Taking on food systems accountability. Agriculture. (2021) 11:422. doi: 10.3390/agriculture11050422

[4] Paris 21. Advancing statistical development in Small Island developing states in the Post-2015 era: the NSDS approach. Paris 21. (2014). Available online at: https://www.paris21.org/sites/default/files/media/document/2023-10/SIDS-PRINT%20v2.pdf (Accessed January 10, 2025)

[5] Sharp MK Andrew NL. (2021). Poverty, malnutrition and food security in Pacific Small Island Developing States. Bangkok: FAO. Available online at: https://openknowledge.fao.org/server/api/core/bitstreams/bd4a44d4-4b5e-4ed6-88f7-e8411e5ebdc1/content? (Accessed January 10, 2025).

[6] Massa I. (2021). Policy brief: food security challenges and vulnerability in small island developing states. Sustainable Development Solutions Network, United Nations. Available online at: https://unsdg.un.org/resources/policy-brief-food-security-challenges-and-vulnerability-small-island-developing-states?utm (Accessed January 10, 2025).

[7] Mohammadi E Singh SJ McCordic C Pittman J Food security challenges and options in the Caribbean: insights from a scoping review Anthr Sci (2022) 1 91–108. doi: 10.1007/s44177-021-00008-8 1

[8] United Nations. United nations data portal. Popul div data portal (2024). Available online at: https://population.un.org/dataportal/data/indicators/49/locations/16,660,533,60,92,136,316,258,580,184,531,312,474,500,540,570,630,663,796,850/start/2024/end/2024/table/pivotbylocation?df=869d454c-ab90-452b-a6ac-a3d0ae907652 (Accessed March 4, 2025).

[9] Kebede EA Abou Ali H Clavelle T Froehlich HE Gephart JA Hartman S. Assessing and addressing the global state of food production data scarcity. Nat Rev Earth Environ. (2024) 5:295–311. doi: 10.1038/s43017-024-00516-2

[10] Ostolaza C Rosas C García-Blanco AM Gittelsohn J Colón-Ramos U. Impact of the COVID-19 pandemic on food insecurity in Puerto Rico. J Hunger Environ Nutr. (2023) 18:380–95. doi: 10.1080/19320248.2021.1997857

[11] Coleman-Jensen A Rabbitt MP Gregory CA Singh A. Household Food Security in the United States in 2021 U.S. Department of Agriculture, Economic Research Service (2022).

[12] Centers for Disease Control and Prevention. BRFSS prevalence and trends data. Centers for Disease Control and Prevention, National Center for Chronic Disease Prevention and Health Promotion, Division of Population Health. (2021). Available online at: https://www.cdc.gov/brfss/data_documentation/index.htm (Accessed April 2, 2024).

[13] Departamento de Salud de Puerto Rico. (2020). Puerto Rico Chronic Disease Action Plan 2014–2020. Available online at: https://www.iccp-portal.org/sites/default/files/plans/Puerto%20Rico%20Chronic%20Disease%20Action%20Plan%20English.pdf (Accessed April 1, 2024).

[14] Akinbami LJ Chen TC Davy O Ogden CL Fink S Clark J. National Health and Nutrition Examination Survey, 2017–March 2020 prepandemic file: sample design, estimation, and analytic guidelines National Center for Health Statistics (2020). 35593699

[15] Buzby JC Bentley J Padera B Campuzano J Ammon C. Updated supermarket shrink estimates for fresh foods and their implications for ERS loss-adjusted food availability data. Washington, DC, United States: USDA Economic Research Service (2016).

[16] USDA, Economic Research Service. Food availability (per capita) data system. (2020). Available online at: https://www.ers.usda.gov/data-products/food-availability-per-capita-data-system/ (Accessed April 1, 2024).

[17] Departamento de Agricultura de Puerto Rico. Ingreso Bruto Agrícola de Puerto Rico. EstadisticasPR (2017). Available online at: https://estadisticas.pr/index.php/en/inventario-de-estadisticas/ingreso-bruto-agricola (Accessed December 5, 2020).

[18] Martínez Mangual M Aponte López LE Vázquez Colón N Hernández Marrero O Velázquez Estrada AL Jara Castro AG. Estadísticas e Índice de Producción Agrícola de Puerto Rico. (2023). Available online at: https://estadisticas.pr/Agricultura (Accessed March 30, 2024).

[19] Schreiber K Hickey G Metson G Robinson B MacDonald G. Quantifying the foodshed: a systematic review of urban food flow and local food self-sufficiency research. Environ Res Lett. (2021) 16. doi: 10.1088/1748-9326/abad59

[20] Griffin T Conrad Z Peters C Ridberg R Tyler EP. Regional self-reliance of the northeast food system. Renew Agric Food Syst. (2015) 30:349–63. doi: 10.1017/S1742170514000027

[21] Stehl J Vonderschmidt A Vollmer S Alexander P Jaacks LM. Gap between national food production and food-based dietary guidance highlights lack of national self-sufficiency. Nat Food. (2025) 6:571–6. doi: 10.1038/s43016-025-01173-4, PMID: 40379972 PMC12185324

[22] World Customs Organization. The harmonized system: a universal language for international trade 30 years on. Belgium: World Customs Organization. (2018). Available online at: https://www.wcoomd.org/-/media/wco/public/global/pdf/topics/nomenclature/activities-and-programmes/30-years-hs/hs-compendium.pdf

[23] USDA and US Department of Health and Human Services. Dietary guidelines for Americans, 2020–2025. 9th ed. (2020). Available online at: https://www.dietaryguidelines.gov/

[24] USDA, Foreign Agricultural Service. (2009). GATS global agricultural trade system “the basics” training manual. Available online at: https://apps.fas.usda.gov/gats/101TheBasics.htm (Accessed April 1, 2024).

[25] Bowman S Clemens J Friday J Moshfegh A. (2020). Food patterns equivalents database 2017–2018: methodology and user guide. Beltsville, MD: Food Surveys Research Group, Beltsville Human Nutrition Research Center; Agricultural Research Service, U.S. Department of Agriculture. Available online at: http://www.ars.usda.gov/nea/bhnrc/fsrg

[26] Junta de Planificación, Gobierno de Puerto Rico. (2020). External trade data. Available online at: http://jp.pr.gov/External-Trade-Data (Accessed April 30, 2024).

[27] USDA, Economic Research Service. Food availability documentation. (2020). Available online at: https://www.ers.usda.gov/data-products/food-availability-per-capita-data-system/food-availability-documentation/ (Accessed April 1, 2024]).

[28] Ariza-Macías J Díaz de Collazo H Sánchez E. Food balance sheet Puerto Rico 1967-1968, a university contribution to assessment of nutritional status. Ecol Food Nutr. (1973) 2:173–80. doi: 10.1080/03670244.1973.9990334

[29] USDA, National Agricultural Statistics Service. 2017 Census of Agriculture Puerto Rico. (2018). Available online at: https://www.nass.usda.gov/Publications/AgCensus/2017/

[30] USDA, National Agricultural Statistics Service (1974) 1974 Census of Agriculture Puerto Rico. Available online at: https://agcensus.library.cornell.edu/census_parts/1974-puerto-rico/

[31] Comas Pagán M. Vulnerabilidad de las cadenas de suministros el cambio climatico y el desarrollo de estrategias de adaptation: El caso de las cadenas de suministros de alimento de Puerto Rico. Doctoral dissertation. Puerto Rico: University of Puerto Rico. (2009). Available online at: https://www.proquest.com/openview/8ef0f3eec1f25f71eaa29c877eac2157/1?pq-origsite=gscholar&cbl=18750&diss=y (Accessed March 30, 2024).

[32] Marrero A Mattei J. Reclaiming traditional, plant-based, climate-resilient food systems in small islands. Lancet Planet Health. (2022) 6:e171–9. doi: 10.1016/S2542-5196(21)00322-3, PMID: 35150626 PMC9031398

[33] Kenner B Russell D Constanza V. Sowell Andrew Pham Xuan Terán Angel. (2023). Puerto Rico’s agricultural economy in the aftermath of Hurricanes Irma and Maria: a brief overview. U.S. Department of Agriculture, Economic Research Service. Available online at: https://www.ers.usda.gov/webdocs/publications/106261/ap-114.pdf

[34] Muimba-Kankolongo A. Chapter 9 - root and tuber crops In: Muimba-Kankolongo A, editor. Food crop production by smallholder farmers in southern Africa. UK and USA: Academic Press (2018). 123–72.

[35] Okogbenin E Setter TL Ferguson M Mutegi R Ceballos H Olasanmi B. Phenotypic approaches to drought in cassava: review. Front Physiol. (2013) 4:93. doi: 10.3389/fphys.2013.00093, PMID: 23717282 PMC3650755

[36] Ferdaus MJ Chukwu-Munsen E Foguel A da Silva RC. Taro roots: an underexploited root crop. Nutrients. (2023) 15:3337. doi: 10.3390/nu15153337, PMID: 37571276 PMC10421445

[37] Gatto M Naziri D San Pedro J Béné C. Crop resistance and household resilience – the case of cassava and sweetpotato during super-typhoon Ompong in the Philippines. Int J Disaster Risk Reduct. (2021) 62:102392. doi: 10.1016/j.ijdrr.2021.102392

[38] Díaz M. (2018). Importación de carne, opaca producción local en Puerto Rico. El Nuevo Día. Available online at: https://www.elnuevodia.com/negocios/consumo/notas/las-importaciones-se-apoderan-del-suplido-de-carnes-en-la-isla/ (Accessed October 16, 2024).

[39] Thomas A Baptiste A Martyr-Koller R Pringle P Rhiney K. Climate change and small island developing states. Annu Rev Environ Resour. (2020) 45:1–27. doi: 10.1146/annurev-environ-012320-083355

[40] Petzold J Magnan AK. Climate change: thinking small islands beyond Small Island developing states (SIDS). Clim Chang. (2019) 152:145–65. doi: 10.1007/s10584-018-2363-3

[41] Hernández JC Comas-Pagan M Jiménez A Blas S. La aportación de la producción local y las importaciones de alimentos en la demanda calórica de Puerto Rico. J Agric Univ PR. (2017) 101:121–41. doi: 10.46429/jaupr.v101i1.14298

[42] Halliday C Morrissey K Saint Ville A Guell C Augustus E Guariguata L. Trends in food supply, diet, and the risk of non-communicable diseases in three Small Island developing states: implications for policy and research. Front Sustain Food Syst. (2023) 7:1058540. doi: 10.3389/fsufs.2023.1058540

[43] Krebs-Smith SM Pannucci TE Subar AF Kirkpatrick SI Lerman JL Tooze JA. Update of the healthy eating index: HEI-2015. J Acad Nutr Diet. (2018) 118:1591–602. doi: 10.1016/j.jand.2018.05.021, PMID: 30146071 PMC6719291

[44] Mattei J Tamez M Bigornia SJ Noel SE Xiao RS Ríos-Bedoya CF. Dietary intake and its determinants among adults living in the metropolitan area of Puerto Rico. Nutrients. (2019) 11:1598. doi: 10.3390/nu11071598, PMID: 31337152 PMC6683066

[45] Colón-López V Banerjee G Gertz AM Ortiz AP Calo W Finney-Rutten LJ. Behavioral correlates of fruit and vegetable intake in Puerto Rico: results from the health information national trends survey. PR Health Sci J. (2013) 32:194–9. Available online at: https://pmc.ncbi.nlm.nih.gov/articles/PMC4994519/PMC4994519 24397217

[46] Truesdell E Schelske-Santos M Nazario CM Rosario-Rosado RV McCann SE Millen AE. Foods contributing to macronutrient intake of women living in Puerto Rico reflect both traditional Puerto Rican and western-type diets. Nutrients. (2018) 10:1242. doi: 10.3390/nu10091242, PMID: 30200564 PMC6163587

[47] Suárez GómezII W. Cabotage as an external non-tariff measure on the competitiveness on SIDS’s agribusinesses: the case of Puerto Rico. Cent J. (2018) 30:172-207.v. Available online at: http://hdl.handle.net/10454/16904

[48] Cuadra CMO Davidson R. Eating Puerto Rico: a history of food, culture, and identity. Chapel Hill, NC, USA: University of North Carolina Press (2013).

[49] Sanjur D. Puerto Rican food habits: a socio-cultural approach. Ithaca, NY. (1970). Available online at: https://babel.hathitrust.org/cgi/pt?id=coo.31924085798712&seq=16 (Accessed July 2, 2025).

[50] Mintz SW. Reflections on Caribbean peasantries. Nieuwe West-Indische Gids New West Indian Guide. (1983) 57:1–17.

[51] Mattei J McClain AC Falcón LM Noel SE Tucker KL. Dietary acculturation among Puerto Rican adults varies by acculturation construct and dietary measure. J Nutr. (2018) 148:1804–13. doi: 10.1093/jn/nxy174, PMID: 30383277 PMC6669953

[52] World Bank. GDP per capita (current US$). World Bank Open Data (2023). Available online at: https://data.worldbank.org/indicator/NY.GDP.PCAP.CD?locations=PR (Accessed February 28, 2025).

[53] United Nations. (2024). SIDS: economic situation and prospects. Available online at: https://www.un.org/development/desa/dpad/wp-content/uploads/sites/45/SIDS_WESP-2024_Mid-Year_Folded-Leaflet_WEB.pdf (Accessed February 28, 2025).

[54] US Census Bureau. “AGE AND SEX” American Community Survey, ACS 1-year estimates subject tables, table S0101. US Census Bur (2016) Available online at: https://data.census.gov/table/ACSST1Y2016.S0101?q=population (Accessed April 1, 2024).

[55] US Census Bureau. “AGE AND SEX” American Community Survey, ACS 1-year estimates subject tables, table S0101. (2017) Available online at: https://data.census.gov/table/ACSST1Y2017.S0101?q=population (Accessed April 1, 2024).

[56] US Census Bureau. “AGE AND SEX” American Community Survey, ACS 1-year estimates subject tables, table S0101. (2018). Available online at: https://data.census.gov/table/ACSST1Y2018.S0101?q=population (Accessed April 1, 2024).

